# Moderately thermostable GH1 β-glucosidases from hyperacidophilic archaeon *Cuniculiplasma divulgatum* S5

**DOI:** 10.1093/femsec/fiae114

**Published:** 2024-08-10

**Authors:** Anna N Khusnutdinova, Hai Tran, Saloni Devlekar, Marco A Distaso, Ilya V Kublanov, Tatiana Skarina, Peter Stogios, Alexei Savchenko, Manuel Ferrer, Olga V Golyshina, Alexander F Yakunin, Peter N Golyshin

**Affiliations:** Centre for Environmental Biotechnology, School of Environmental and Natural Sciences, Bangor University, Bangor, LL57 2UW, United Kingdom; Centre for Environmental Biotechnology, School of Environmental and Natural Sciences, Bangor University, Bangor, LL57 2UW, United Kingdom; Centre for Environmental Biotechnology, School of Environmental and Natural Sciences, Bangor University, Bangor, LL57 2UW, United Kingdom; Centre for Environmental Biotechnology, School of Environmental and Natural Sciences, Bangor University, Bangor, LL57 2UW, United Kingdom; Department of Plant Pathology and Microbiology, Faculty of Agriculture, Food and Environment, Hebrew University of Jerusalem, Rehovot, 7610001, Israel; Department of Chemical Engineering and Applied Chemistry, University of Toronto, Ontario, M5S 3E5, Canada; Department of Chemical Engineering and Applied Chemistry, University of Toronto, Ontario, M5S 3E5, Canada; Department of Microbiology Immunology and Infectious Diseases, University of Calgary, Calgary, Alberta, T2N 1N4, Canada; Departamento de Biocatalisis Aplicada, Instituto de Catalisis y Petroleoquimica (ICP), CSIC, Madrid, 28049, Spain; Centre for Environmental Biotechnology, School of Environmental and Natural Sciences, Bangor University, Bangor, LL57 2UW, United Kingdom; Centre for Environmental Biotechnology, School of Environmental and Natural Sciences, Bangor University, Bangor, LL57 2UW, United Kingdom; Centre for Environmental Biotechnology, School of Environmental and Natural Sciences, Bangor University, Bangor, LL57 2UW, United Kingdom

**Keywords:** acidic environments, acidophilic archaea, β-glucosidase, cellobiohydrolase, *Cuniculiplasma*, extremozymes, GH1, Thermoplasmatales

## Abstract

Family GH1 glycosyl hydrolases are ubiquitous in prokaryotes and eukaryotes and are utilized in numerous industrial applications, including bioconversion of lignocelluloses. In this study, hyperacidophilic archaeon *Cuniculiplasma divulgatum* (S5^T^=JCM 30642^T^) was explored as a source of novel carbohydrate-active enzymes. The genome of *C. divulgatum* encodes three GH1 enzyme candidates, from which CIB12 and CIB13 were heterologously expressed and characterized. Phylogenetic analysis of CIB12 and CIB13 clustered them with β-glucosidases from genuinely thermophilic archaea including *Thermoplasma acidophilum, Picrophilus torridus, Sulfolobus solfataricus, Pyrococcus furiosus*, and *Thermococcus kodakarensis*. Purified enzymes showed maximal activities at pH 4.5–6.0 (CIB12) and 4.5–5.5 (CIB13) with optimal temperatures at 50°C, suggesting a high-temperature origin of *Cuniculiplasma* spp. ancestors. Crystal structures of both enzymes revealed a classical (α/β)_8_ TIM-barrel fold with the active site located inside the barrel close to the C-termini of β-strands including the catalytic residues Glu204 and Glu388 (CIB12), and Glu204 and Glu385 (CIB13). Both enzymes preferred cellobiose over lactose as substrates and were classified as cellobiohydrolases. Cellobiose addition increased the biomass yield of *Cuniculiplasma* cultures growing on peptides by 50%, suggesting that the cellobiohydrolases expand the carbon substrate range and hence environmental fitness of *Cuniculiplasma*.

## Teaser

Novel β-glucosidases expand growth substrate spectrum in peptidolytic *Cuniculiplasma divulgatum* (Thermoplasmatales).

## Introduction

Extremophiles thriving in environments with physico–chemical conditions hostile to common microorganisms, are widely distributed across the globe. Archaea often outcompete bacteria and eukaryotes in extreme environments, and flourish at high temperatures, at low or high pH values and at elevated salinities (Shu and Huang 2022). They employ various and often unique physiological properties, which result in products of biotechnological importance useful for applications in different industries (Elleuche et al. 2014). One of such organisms is *Cuniculiplasma divilgatum*, ubiquitous in moderate-to-low temperature acid mine drainage systems, with pH optimum at 1.0–1.2 (Golyshina et al. 2016a,b). *Cuniculiplasma* spp. are also found in geothermal areas worldwide, pointing at their global distribution in acidic environments of different origin and at variety of temperature adaptations in these archaea (Golyshina et al. 2019). Taxonomically, the genus *Cuniculiplasma* is included in the order *Thermoplasmatales*, which contains organisms with the lowest pH values for growth ever recorded (Golyshina et al. 2019). Potentially, *Cuniculiplasma* spp. can serve as a source of extremozymes, enhancing our comprehension of these enzymes’ functions and their significance in the life strategies and ecology of extremophilic archaea, while also offering promise for potential biotechnological uses.

*Cuniculiplasma divulgatum* S5 genome encodes 22 glycoside hydrolases (GHs), according to CAZY records (http://www.cazy.org/a7595.html; Cantarel et al. 2009), three of which belong to the Glycosyl Hydrolases Family 1 (GH1) that encompasses a large set of enzymes sharing a catalytic domain with a (α/β)8 TIM-barrel fold and a retaining mechanism of catalysis with activities spread across 34 EC numbers (CAZY database; Cantarel et al. 2009). These enzymes are ubiquitous across all domains of life and play fundamental roles, such as *in vivo* degradation of lignocellulosic materials for nutrient uptake. Additionally, they have a wide range of *in vitro* applications in food, medicine, and the production of bio-based chemicals and renewable energy sources (Cantarel et al. 2009, Ketudat Cairn and Esen 2010). GH1 enzymes are encoded in genomes of archaea, with 25 enzymes functionally characterized, including from two taxonomic neighbours of *C. divulgatum, Thermoplasma acidophilum* DSM 1728 (Kim et al. 2009), and from *Picrophilus torridus* DSM 9790 (Murphy and Walsh 2019)*. Cuniculiplasma divulgatum* encodes three such GH1 proteins, one of which was partially characterized, however, only with model, *p*-nitrophenyl (*p*NP) glucoside substrate, with no activity determined against natural substrates (He et al. 2023).

*Cuniculiplasma* spp. are known (Golyshina et al. 2016a,b), like their closest relatives from *Thermoplasmatales* (Huber and Stetter 2006), to rely on scavenging detritus/dead cell biomass of microorganisms, and correspondingly, their growth media contain complex organic peptide-containing ingredients, such as yeast, meat, or beef extract, tryptone, or peptone. They occupy niches with other related *Thermoplasmatales*, but also, intriguingly, with primary producing, photosynthetic organisms, such as *Chlamydomonas acidophila* or *Euglena mutabilis* (Distaso et al. 2022) that supply polysaccharides in the environment. It is known that monomeric sugars did not enhance growth of *C. divulgatum*, however, the ability of *Cuniculiplasma* spp. to make use of other products/intermediates of polysaccharide hydrolysis, remains to be assessed.

The aim of present study was therefore to heterologously express, purify, and structurally and functionally characterize, the GH1 carbohydrate-active enzymes from *C. divulgatum*. Moreover, to assess the ecological importance of these enzymes for *Cuniciliplasma* spp. in their natural habitats, we examined the enzyme substrates for their ability to support the growth of these microorganisms.

In this work, we report the results of our study on two β-glucosidases CIB12 and CIB13 with respect to their phylogeny, biochemical properties, and substrate specificities with model and natural substrates, accompanied with the analysis of their resolved crystal structures. These intracellular enzymes have highest activities towards cellobiose, amendment of which to the medium significantly increased the biomass yield of *C. divulgatum*, pointing at physiological and ecological importance of these GH1 family cellobiohydrolases.

## Materials and methods

### Strains, culture conditions, and plasmids

*Cuniculiplasma divulgatum* (S5^T^=JCM 30642^T^) from Cantareras acid mine drainage site in Spain was formally described earlier (Golyshina et al. 2016a). The modified medium DSMZ 88 was used for cultivation of the strain S5, which contained (g l^−1^): (NH_4_)_2_SO_4_, 1.3; KH_2_PO_4_, 0.28; MgSO_4_ ^.^7H_2_O, 0.25; CaCl_2_^.^2H_2_O, 0.07; and FeCl_3_^.^6H_2_O, 0.02. The medium was also supplemented with the trace element solution SL-10 from DSMZ medium 320 in proportion 1:1000 (v/v), betaine at 0.06% (w/v), and vitamin solution Kao and Michayluk (Sigma-Aldrich, Gillingham, UK) at 1:100 (v/v). As the primary carbon source, the beef extract (ThermoFisher Scientific, Paisley, UK) was added at final concentration 3 g l^−1^. In addition, cellobiose and lactose (Sigma-Aldrich and Scientific Laboratory Supplies, Nottingham, UK, respectively) were tested as carbon sources at concentration of 3 mM (0.1% w/v). The pH of the medium was adjusted to 1.0 with concentrated H_2_SO_4_. Cultivation was conducted at 37°C in Erlenmeyer flasks in an orbitary shaker at 100 r m^−1^. The growth was followed by measuring the culture optical density at 600 nm using a BioPhotometer Plus (Eppendorf, Hamburg, Germany). Consumption of added lactose and cellobiose during the growth of *C. divulgatum* was measured by HPLC analysis of culture supernatants using a Shimadzu Prominence-I LC-2030c 3D Plus instrument. Samples of growth media were centrifuged at relative centrifugal force (rcf) of 10 000 for 10 min, filtered through nylon filters (0.22 µm), and 10 µl-aliquots of cleared samples were used for HPLC analysis. Cellobiose was analysed using a Bio-Rad Aminex 87H columns at 50°C (mobile phase 5 mN H_2_SO_4_, 0.6 ml min^−1^), whereas lactose was measured using an Aminex 87P column at 80°C (mobile phase H_2_O, 1 ml min^−1^).

*Escherichia coli* strains used in this study were cultivated on Luria–Bertani (LB) medium (per litre of deionised water, 10 g tryptone, 5 g yeast extract, and 5 g NaCl) with 15 g l^−1^ agar amended in solid media, at 37°C. Strains harbouring the recombinant plasmids were grown in LB medium with ampicillin at the final concentration of 100 µg ml^−1^ (Li et al. 2021).

Genes encoding the selected GH1 candidates, CIB12 (GenBank accession number SIM59545.1, locus tag: CSP5_0942) and CIB13 (SIM79973.1, pol CSP5_1651) were amplified by polymerase chain reaction (PCR) from *C. divulgatum* S5 genomic DNA and cloned into the p15TvL protein expression plasmid (Novagen/Merck, Darmstadt, Germany) containing an N-terminal 6His-tag as described previously; same genes were partially codon-optimized for *E. coli* and synthesized at Twist Biosciences alongside their mutant variants, H148A, N203A, E204A, Y321A, Y341A, W362A, E388A, and W426A for CIB12 and W432A, N203A​, E204A​, Y320A​, Y340A​, W359A​, N148A​, and W426A. Recombinant plasmids were initially transformed into the nonexpression *E. coli* DH5α host (Invitrogen, Carlsbad, CA, USA), from which, after overnight growth they were extracted and transformed into *E. coli* BL21(DE3) Lobstr® (Kerafast, USA) expression host used for recombinant protein production and purification.

### Protein expression and purification

20-ml starter cultures of *E. coli* BL21(DE3) Lobstr® harbouring recombinant plasmids were grown on a shaker at 37°C for 18 h in LB medium supplemented with 100 µg ml^−1^ ampicillin, transferred into 1 l of 0.5x Terrific Broth medium (Sambrook et al. 1989) containing 0.4% (v/v) glycerol and ampicillin (100 µg ml^−1^), and cultured at 37°C in baffled flasks in a shaking incubator (250 r m^−1^). When the optical density (600 nm) reached 0.6–0.8 units, 0.4 mM isopropyl-β-d-thiogalactopyranoside was added and incubation continued overnight at 16°C with shaking at 250 r m^−1^. Cells were harvested by centrifugation at 4000 × *g* (15 min at 20°C) and resuspended in 20 ml of binding buffer (50 mM 4-(2-hydroxyethyl)-1-piperazineethanesulfonic acid (HEPES), pH 7.5, 500 mM NaCl, 5 mM imidazole, 5% glycerol). Cells were disrupted by sonication (300 W, 3 s strokes, and 4 s intervals, for 10 min) in an ice bath, and clear lysates were obtained after centrifugation at 40 000 × *g* (30 min at 4°C). Recombinant proteins were purified from lysates by metal-affinity chromatography using Ni-NTA His-Bind Resin (Merck). The lysates were loaded on gravity columns equilibrated with 10 ml of binding buffer (50 mM HEPES, pH 7.5, 500 mM NaCl, 5 mM imidazole, 5% glycerol) and washed with 10 ml of washing buffer (50 mM HEPES, pH 7.5, 500 mM NaCl, 20 mM imidazole, 5% glycerol) to remove nonspecifically bound proteins. Bound recombinant proteins were eluted with an elution buffer (50 mM HEPES, pH 7.5, 500 mM NaCl, 200 mM imidazole, 5% glycerol). Protein concentration was determined using a Bradford reagent at 595 nm in a 96-well plate with BSA as a standard. Purified proteins were frozen in droplets in liquid nitrogen and stored at −80°C (Li et al. 2021). The purity and apparent size of purified proteins were analysed by denaturing polyacrylamide gel electrophoresis (SDS-PAGE) using the precast RunBlue 10% gels (Expedeon, Harston, UK). The standard protein markers (10–250 kDa, Thermo Scientific) were used to estimate the apparent molecular weight of expressed proteins.

### GH assays with chromogenic and natural substrates

GH activity of purified GH1 proteins was assayed using two panels of chromogenic (model) and natural GH substrates (Tables S1 and S2). The reaction mixtures contained 20 mM MES buffer (pH 5.0), 1 mM *p*NP-glycosyl substrate, and 3 µg of enzyme in the final volume of 200 µl (incubation at 37°C for 120 min). The absorbance of the released *p*-nitrophenol was measured at 410 nm. One unit (U) of enzyme activity was defined as the amount of enzyme needed to liberate 1 µmol of *p*NP per minute under the assay conditions. The specific activity of enzyme referred to the number of units of enzyme activity per mg of protein. Reaction mixtures for assays with natural substrates (0.2 ml) contained 20 mM MES buffer (pH 6.0), 1 mM substrate, and 3 µg of purified enzyme. After overnight incubation at 30°C, 10-µl aliquots of reaction mixtures were used to determine the concentration of released reducing sugars using a modified bicinchoninic acid (BCA) assay (Millipore, Gillingham, UK) by adding 10 µl of 2 M NaOH and 200 µl of fresh BCA reagent mix. After 30 min incubation at 60°C in a shaker (500 r m^−1^), the solution absorbance was measured at 562 nm. A standard curve was prepared by plotting the average blank–measurement for each reducing sugar monomer concentration used to calculate the released monomer concentration in reactions (Li et al. 2021). GH activity of purified wild-type and mutant enzymes was determined under indicated reaction conditions using 2 mM *p*NP-β-d-glucopyranoside or 25 mM cellobiose as substrates. All assays were performed in triplicate, and the results are presented as means ± SD (standard deviations) from at least two independent experiments. One unit (U) of enzyme activity was defined as the amount of enzyme required to produce 1 µmol of *p*NP per minute under the assay conditions.

### Kinetic parameters

Michaelis–Menten constant (*K_M_*) and maximum reaction velocity (*V_max_*) of purified CIB12 and CIB13 were determined by assaying enzymes activity in the presence of increasing concentrations of substrates: *p*NP-β-d-glucopyranoside (0–10 mM), cellobiose or lactose (0–150 mM) in 50 mM MES buffer (pH 5.0) using 3 µg or 5 µg of enzyme, respectively, per 0.2 ml reaction. For assays with *p*NP-β-d-glucopyranoside as substrate (30 min incubation at 30°C), the absorbance of released *p*NP was measured at 410 nm after adding 10 µl of 1 M Tris–Cl buffer (pH 9.0) for complete colour development. For assays with natural substrates, the reaction mixtures were incubated at 30°C for 4 h and then filtered using centrifugal filters (PES, 10 kDa cut-off, VWR, 10 000 × *g*). 10 µl aliquots of filtrate were used for the HPLC analysis of released glucose using an Aminex 87H column (60°C, mobile phase 5 mN H_2_SO_4_, isocratic flow at 0.6 ml min^−1^, RI detector). The standard curve was used to determine glucose concentrations in samples and then calculate enzyme activity (µmol min^−1^ mg^−1^). The obtained values were fitted to the classical Michaelis–Menten equation, and the values for *V_max_, k_cat_, K_M_*, and catalytic efficiency (*k_cat_*/*K_M_*) were calculated using a GraphPad Prism software (Swift 1997, Li et al. 2021).

### Analysis of optimal reaction conditions (pH, temperature, NaCl, and Tween-20)

The pH dependency of GH activities of CIB12 and CIB13 was investigated by incubating the purified enzymes (3 µg per 0.2 ml reaction mixture) in 20 mM Britton–Robinson pH buffer (pH 3.0–12.0) at 30°C for 120 min with 2 mM *p*NP-β-d-glucopyranoside as substrate. After incubation, 10-µl aliquots of 1 M Tris–HCl buffer (pH 9.0) were added to each assay for complete colour development and produced *p*-nitrophenol (*p*NP) was determined spectrophotometrically at 410 nm. Temperature profiles of GH activity of purified CIB12 and CIB13 with 2 mM *p*NP-β-d-glucopyranoside as substrate were measured at temperatures 25°C–90°C using 3 µg of protein per reaction in 50 mM MES buffer (pH 5.0). Reaction mixtures were incubated at indicated temperatures for 120 min, and the absorbance of the released *p*NP was measured at 410 nm. The effect of NaCl concentrations (0–4.0 M) on enzymatic activity of CIB12 and CIB13 (3 µg of protein per reaction) was determined using 25 mM cellobiose as substrate (due to high background observed with *p*NP-β-d-glucopyranoside in the presence of high NaCl concentrations). Reaction mixtures (0.2 ml) contained 50 mM MES buffer (pH 5.0), 25 mM cellobiose, and 3 µg of protein (incubation for 240 min at 30°C). The effect of Tween-20 (a detergent) on enzymatic activity of CIB12 and CIB13 was investigated using reaction mixtures (0.2 ml) containing 50 mM MES buffer (pH 5.0), 1 mM *p*NP-β-d-glucopyranoside, and 3 µg of protein (incubation for 120 min at 30°C).

### Analysis of enzyme thermostability and denaturation temperature

Purified proteins were incubated at different temperatures ranging from 30°C to 80°C for 1–5 h, and the residual enzyme activity was measured at 30°C using *p*NP-β-d-glucopyranoside as substrate (8.9 mM for CIB12 and 1.8 mM for CIB13) in 20 mM HEPES buffer (pH 7.0) for CIB12 or in 20 mM MES buffer (pH 6.0) for CIB13, 3 µg of enzyme in 200 µl reaction mixtures. Protein melting temperatures of purified enzymes were determined using differential scanning fluorimetry (DSF) with Sypro Orange® (Invitrogen, Thermo Fisher Scientific, USA) as a reporter dye (Elgert et al. 2020). Samples were loaded into 96-well optically transparent plates (Bio-Rad, USA), and the heating rate was 1.0°C min^−1^ with fluorescence readings (excitation at 530 ± 30 nm, emission at 575 ± 20 nm) taken after each 1° increase. The reaction mixtures containing 10 µg of enzyme, 25 X Sypro Orange® dye and 20 mM buffer (for CIB12: HEPES buffer, pH 7.0; CIB13: MES buffer, pH 6.0) were mixed and heated from 25°C–95°C in increments of 1°C min^−1^ on a CFX96 Real-Time System-C1000 thermal cycler (Bio-Rad). All measurements were done in triplicate. SYPRO Orange dye interacts with a protein undergoing thermal unfolding, with its fluorescence increasing upon exposure to the protein’s hydrophobic core. The *T*_m_ for CIB12 and CIB13 were determined as the melting temperature that correlates with half of the maximal fluorescence signal from data plotted using Boltzmann equation in Graph Pad Prizm 6.0.

### Protein crystallization and crystal structure determination

Purified CIB12 and CIB13 (protein concentrations 10 mg ml^−1^) were crystallized at room temperature using the sitting-drop vapour diffusion method. The reservoir solutions used were 25% PEG 3350, 0.2 M sodium tartrate, 0.1 M Tris (pH 8.5), 0.3 M NDSB 201 (nondetergent sulfobetaines), and 1% 1-butyl-2,3-dimethylimidazolium tetrafluoroborate for CIB12, and 25% PEG3350, 0.2 M KCl, and 0.1 M Tris (pH 9.0) for CIB13. Crystals were cryoprotected by transferring into paratone oil and flash frozen in liquid nitrogen.

Diffraction data was collected at 100 K on a Rigaku home source Micromax-007 with R-AXIS IV++ detector and processed using HKL3000 (Minor et al. 2006). Both CIB12 and CIB13 structures were solved by molecular replacement using Phenix.phaser (Liebschner et al. 2019), and models were generated by the Phyre2 server (Kelley et al. 2015) onto the structure of a β-glucosidase from *Pyrococcus furiosus* (PDB code 3APG). Model building and refinement were performed using Phenix.refine and Coot (Emsley and Cowtan 2004). Translation/Libration/Screw (TLS) parameterization was utilized for refinement and *B*-factors were refined as isotropic. Structure geometry and validation were performed using Phenix Molprobity tools. Data collection and refinement statistics for both structures are summarized in Table S3. The structures were deposited to the Protein Data Bank with PDB accession codes 8U7F (CIB12) and 8U7G (CIB13).

### Bioinformatic analyses

Multiple amino acid sequence alignments were produced using Muscle MAFFT online, phylogenetic trees were constructed in Geneious Prime (Biomatters Ltd., New Zealand) and visualized in ItolTree (www.itol.embl.de).

The phylogenetic tree was built using neighbour-joining method (Saitou and Nei 1987), evolutionary distances were computed using the Poisson correction method (Zuckerkandl and Pauling 1965), for bootstrap analysis, 1000 tree pseudoreplicates were used (Felsenstein 1985), all of which are a part of MEGA X package (Kumar et al. 2018). 3D protein structures were analysed using Chimera X v.1.4 and PyMOL (the PyMOL Molecular Graphics System, version 3.0 Schrodinger, LLC).

## Results and discussion

### CIB12 and CIB13 cocluster with homologues from *Thermoplasmatota* and *Thermococcota*

**Figure 1.**
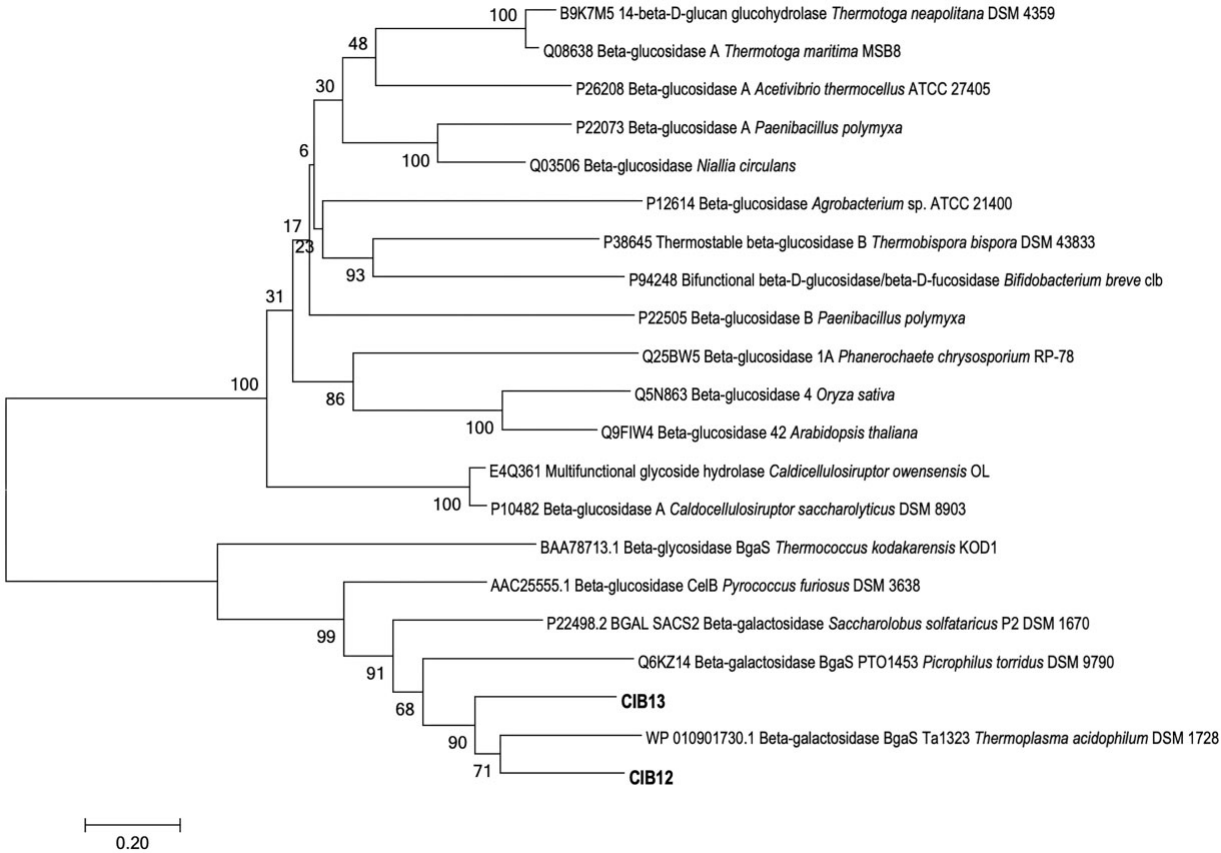
A Neighbour-joining phylogenetic tree of CIB12 and CIB13 with their functionally and/or structurally characterized GH1 family homologues. Sequences were aligned using Muscle (Edgar 2004), bootstrap values were calculated with 1000 pseudoreplicate trees, Poisson substitution model was used. Evolutionary analyses were conducted in MEGAX (Kumar et al. 2018). Scale bar, 0.2 substitutions per position.

Currently, 186 families of glycosyl hydrolases are classified, and they are grouped into 16 different superfamilies and added clans (Shrivastava 2020). To classify the CIB12 and CIB13, they were screened against InterPro database and identified as GH1 family of enzymes. Both enzymes belong to IPR017853 superfamily of glycosyl hydrolases, more specifically to GH1 family, represented in InterPro database with IPR001360 family. In Pfam database these enzymes belong to PF00232 domain proteins, with protein fingerprints PR00131 in PRINTS database. Glycosyl hydrolases in the GH1 family are ubiquitously distributed across the tree of life, appearing in both eukaryotic and prokaryotic organisms due to their diverse substrate specificities. In IPR001360 family, total number of species are 16 034, where bacteria account for 13 216 representatives, eukaryotic species for 2544 and archaeal species for 250 (https://www.ebi.ac.uk/interpro/entry/InterPro/IPR001360). To establish the phylogenetic affiliation of CIB12 and CIB13 with characterized glycosyl hydrolases, initially, 260 sequences referenced as “reviewed” in the UniProt database were used (UniProt Consortium 2019). The majority of branches within the tree encompass sequences of eukaryotic glycosyl hydrolases sourced from *Homo sapiens* as well as model plants *Arabidopsis thaliana* and *Oryza sativa* (Fig. S1A). Interestingly, two distinct branches have only prokaryotic sequences of GH1 family, in contrast to five branches having solely eukaryotic sequences, though as previously mentioned, eukaryotic enzymes are less presented, but more studied. Figure 1 provides a more detailed view of phylogenetic placement of CIB12 and CIB13 and their closest homologues. CIB12 and CIB13 share 55% amino acid sequence identity between themselves, their further functionally and/or structurally characterized top homologues were *Thermoplasmatales*-derived BgaS from *T. acidophilum* DSM 1728 (61/54% sequence identity) (Kim et al. 2009), and β-galactosidase (BgaS) from *P. torridus* DSM 9790 (46/46%) (Murphy and Walsh 2019). Further homologues from the same monophyletic cluster were derived from other truly thermophilic archaea, β-galactosidases from *Acidilobus saccharovorans* (44% and 47%) (Trofimov et al. 2013), *Saccharolobus solfataricus* P2 (44% and 47%) (Cubellis et al. 1990), and β-glucosidase CelB from *P. furiosus* (44% and 46%) (Li et al. 2013), β-glucosidase BglB from *Thermosphaera aggregans* (46% and 46%) (Chi et al. 1999) and *Thermococcus kodakarensis* KOD1 (38% and 40%) (Hwa et al. 2015). Amino acid sequence alignment suggested that Glu204 and Glu388 represent the catalytic glutamate residues of CIB12, whereas Glu204 and Glu385 are present in CIB13. As expected, these amino acid residues are highly conserved in all GH1 members.

### CIB12 and CIB13 are GH1 GHs with a preference towards cellobiose

In this study, CIB12 and CIB13 were recombinantly expressed and purified to homogeneity by Ni-affinity chromatography as described in the section “Materials and methods”. SDS-PAGE analysis of purified protein samples revealed the presence of one major band corresponding to CIB12 and CIB13 (Fig. S2). The apparent molecular masses of purified recombinant proteins were estimated to be ~56 kDa, which are close to the expected protein sizes.

**Figure 2.**
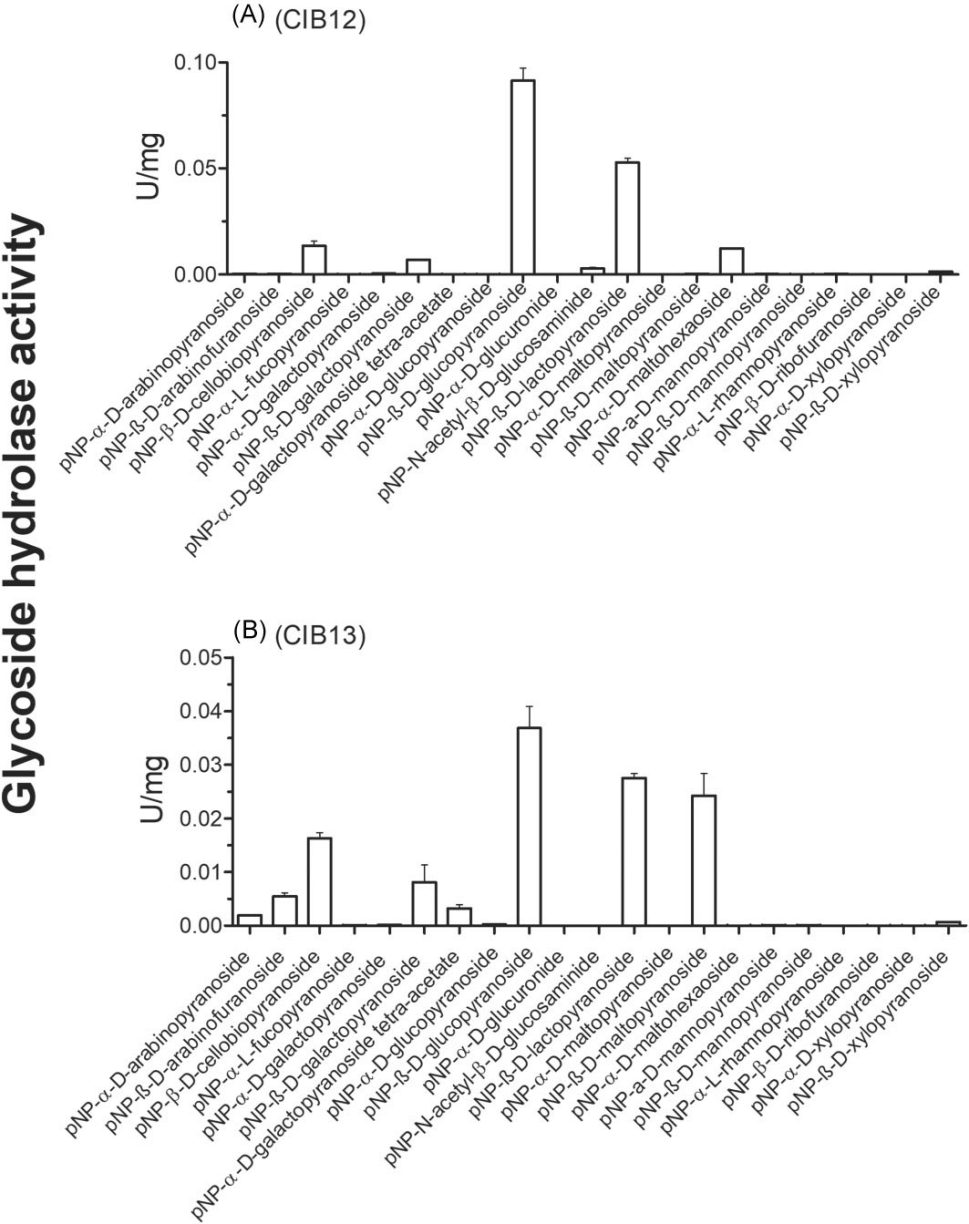
GH activity assays of CIB12 (A) and CIB13 (B) for against 21 chromogenic GH substrates. CIB12 and CIB13 (3 µg for each) were incubated with indicated *p*NP-substrates (1 mM each) at 37°C for 2 h, and the released reaction product *p*-nitrophenol was measured at 410 nm.

**Figure 3.**
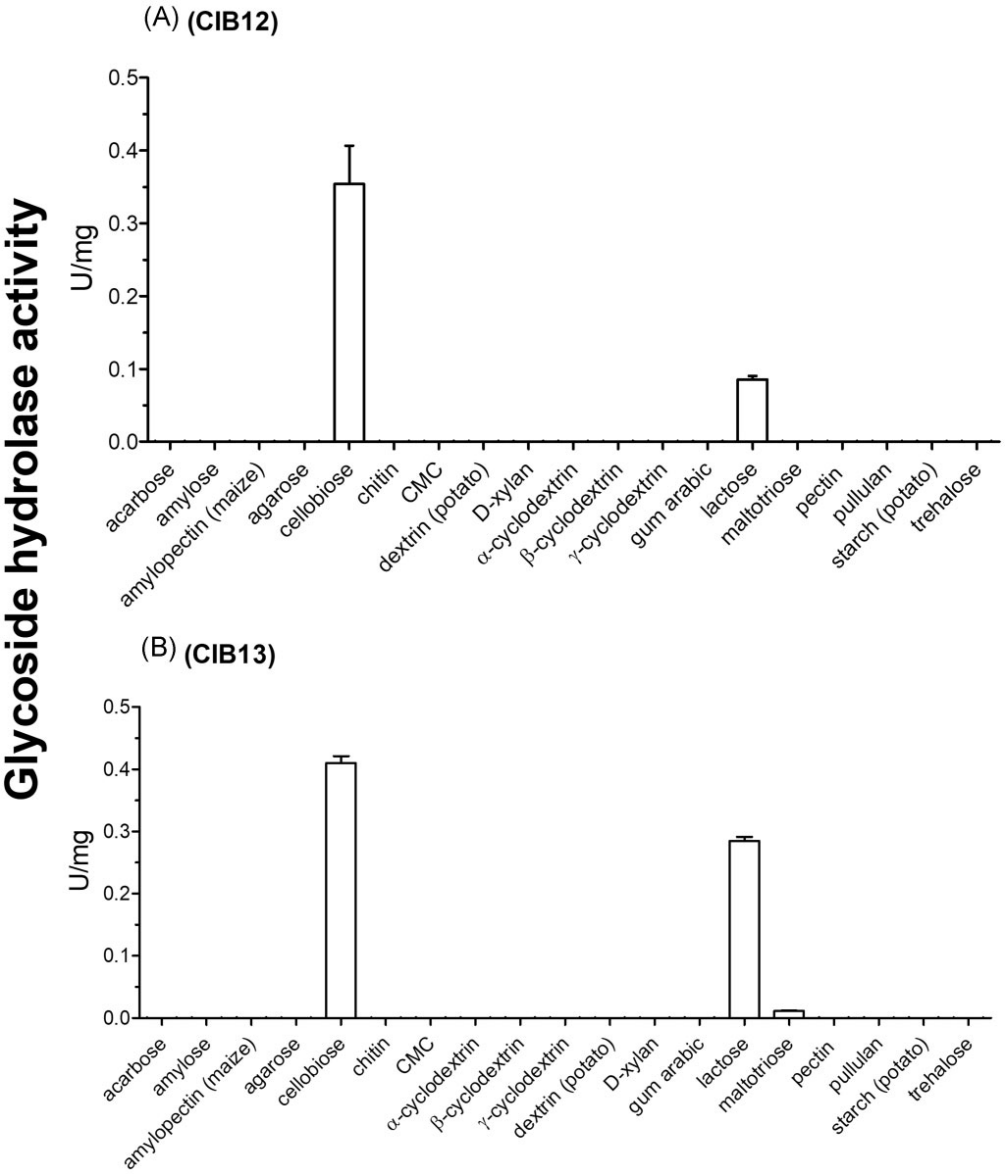
Hydrolytic activity of CIB12 (A) and CIB13 (B) against natural GH substrates. Purified CIB12 and CIB13 (3 µg each) were incubated with 1 mM substrates overnight at 30°C, and 10 µl aliquots of reaction mixture were used for the analysis of produced reducing sugars using a modified BCA assay. The results are means ± SD from at least two independent experiments.

According to the CAZy and BRENDA databases, biochemically characterized GH1 GHs were active against a broad range of natural and chromogenic (*para*-nitrophenyl, *p*NP) substrates, such as *p*NP-β-d-glucopyranoside and *p*NP-β-d-galactopyranoside. Therefore, purified CIB12 and CIB13 were first screened for hydrolytic activity against a panel of 21 chromogenic *p*NP-substrates (Fig. 2). Both CIB12 and CIB13 showed significant activity with five model substrates: *p*NP-β-d-glucopyranoside, *p*NP-β-d-lactopyranoside, *p*NP-β-d-cellobiopyranoside, and *p*NP-β-d-galactopyranoside, whereas CIB13 was also active against *p*NP-α-d-arabinopyranoside and *p*NP-α-d-maltopyranoside, *p*NP-β-d-arabinofuranoside, and *p*NP-α-d-galactopyranoside tetra-acetate (Fig. 2). CIB12 in contrast with CIB13 showed preference towards longer chain, *p*NP-α-d-maltohexaoside versus *p*NP-α-d-maltopyranoside preferred by CIB13. For both proteins, the highest activity was observed with *p*NP-β-d-glucopyranoside, and most of their positive substrates were in the β-d form, validating that CIB12 and CIB13 are β-glucosidases. When screened against a panel of 19 natural GH substrates, both CIB12 and CIB13 were found to be active with two disaccharide substrates, cellobiose and lactose (Fig. 3). Hydrolytic activities of these proteins against cellobiose and lactose are aligned with their substrate preference to *p*NP-β-d-glucopyranoside, *p*NP-β-d-lactopyranoside, and *p*NP-α-d-cellobiopyranoside (Fig. 2). Most of the biochemically characterized GH1 GHs from archaea and bacteria were reported to be active against *p*NP-β-d-glucopyranoside and *p*NP-β-d-galactopyranoside, whereas hydrolytic activity towards cellobiose and lactose was demonstrated primarily for enzymes from bacteria [e.g. *Exiguobacterium antarcticum, Bacillus subtilis* (Liu et al. 2023), *Humicola grisea* var. *thermoidea* (Nascimento et al. 2010), fungi (*Sporotrichum pulverulentum*; Deshpande and Eriksson 1988), and *Thermochaetoides thermophila* (Lusis and Becker 1973)], as well as for archaea [e.g. *S. solfataricus* (D’Auria et al. 1996), *P. furiosus* (Kim and Ishikawa 2010), *T. kodakaraensis* (Ezaki et al. 1999), *P. torridus* (Murphy and Walsh 2019), and *Thermofilum pendens* (Chen et al. 2021)]. Since both CIB12 and CIB13 exhibited the highest activity with *p*NP-β-d-glucopyranoside and cellobiose, these substrates were used for the biochemical characterization of these enzymes and reaction conditions.

### Optimal pH for GH activity of CIB12 and CIB13 is weakly acidic

**Figure 4.**
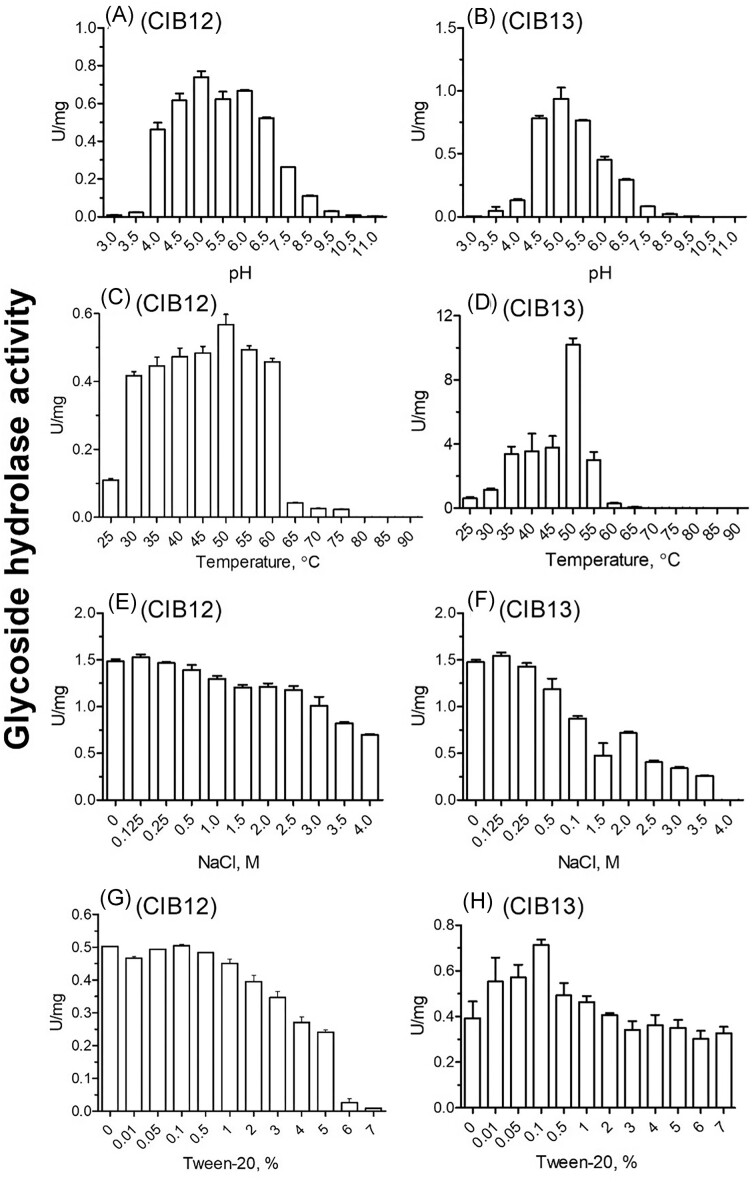
GH activity of purified CIB12 and CIB13 as a function of pH (A and B), temperature (C and D), NaCl concentration (E and F), and Tween-20 concentration (G and H). GH activity of proteins was determined using 2 mM *p*NP-ß-d-glucopyranoside (A, B, C, D, G, H: 3 µg of protein/reaction, 2 h incubations) or 25 mM cellobiose (E, F: 5 µg of protein/reaction, 4 h incubations) as substrates in 50 mM MES buffer (pH 5.0) at 30°C (or as indicated on graphs).

pH-dependency of CIB12 activity was investigated in the range of pH from 3.0 to 12.0. CIB12 was found to be active over a broad pH range from 4.0 to 8.5 (Fig. 4A). The enzyme activity was maximal between pH 4.5 and 6 and was >50% at pH 4.0 and 6.5. pH-dependency of CIB13 was also investigated in the same pH range, 3.0–12.0. CIB13 was active in a narrower pH range. As shown in Fig. 4(B), the optimal pH (pH_opt_) of CIB13 was at 5.0, and it retained at least 75% activity at pH 4.5 and 5.5, which is consistent with other characterized β-glucosidases exhibiting their highest activities within the acidic pH range, typically between pH 4 and 6.5 and having lower activity and may become unstable when exposed to mildly alkaline conditions. CIB12 and CIB13 exhibited pH_opt_ for activities within the same range as their characterized counterparts from various thermoacidophilic archaea: *P. furiosus* (Voorhorst et al. 1995) with a pHopt of 5.5, *P. torridus* with a pH_opt_ of 5.0–5.5 (Murphy and Walsh 2019), *S. solfataricus* P2 with a pH_opt_ of 5.5, and *T. acidophilum* with a pH_opt_ of 6.0 (Kim et al. 2009). CIB12 and CIB13 are derived from the hyperacidophilic organism *C. divulgatum* S5, which optimally grows at pH 1.0–1.2 (Golyshina et al. 2016b). However, both enzymes have a low probability of containing signal peptides, suggesting that they are likely intracellular (nonsecreted) proteins. In that context, noteworthy are earlier records on reduced cytoplasmic pH values (4.6) in *Thermoplasmatales* members, such as *Picrophilus* spp. (Van De Vossenberg et al. 1998), which may explain lower pH optima of intracellular enzymes.

### Optimal temperatures for GH1 activities of CIB12 and CIB13 are higher than those *in situ*

**Figure 5.**
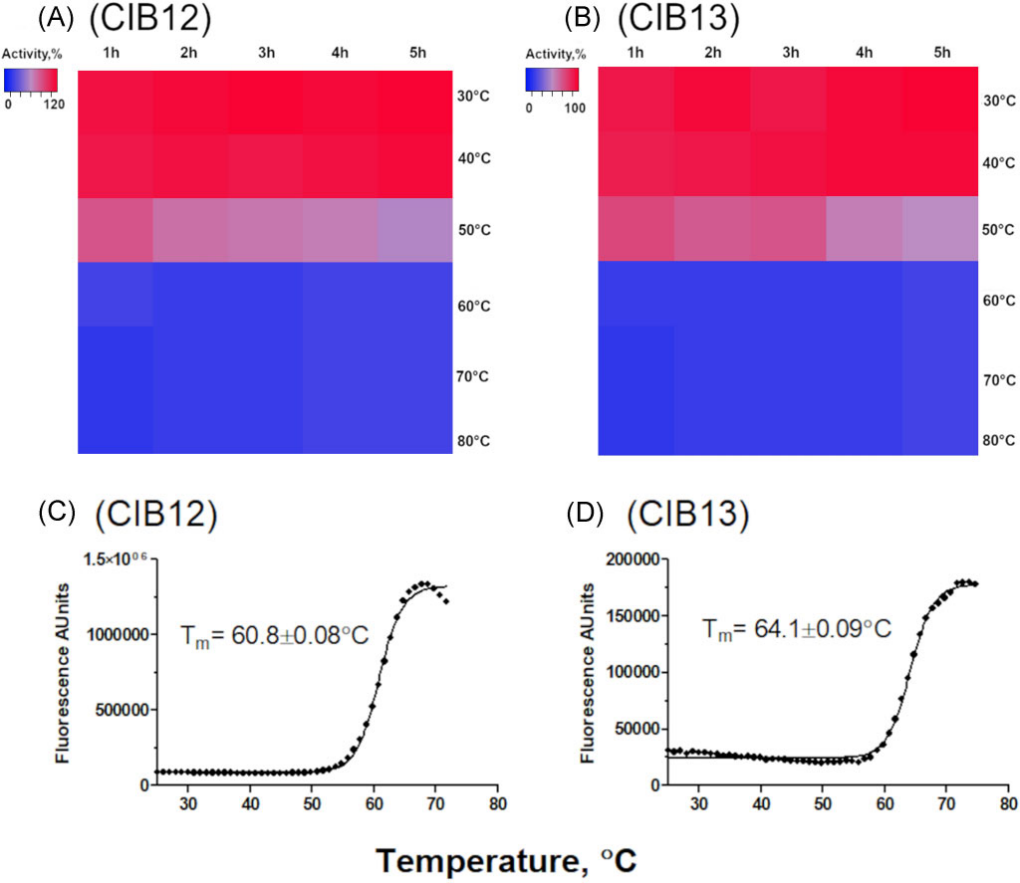
Thermostability of CIB12 and CIB13: activity-based (A and B) and fluorescence (C and D) analyses. (A and B) Purified proteins were incubated at indicated temperatures (30°C–80°C) for different time (1–5 h), and the remaining enzyme activity was measured at 30°C using 2 mM *p*NP-β-d-glucopyranoside as substrate. (C and D), Determination of protein melting point (*T*m) of enzymes by differential scanning fluorescence (DSF) using SYPRO Orange dye. Temperature-induced protein unfolding was monitored in real-time using 10 µg of protein/sample (0.2 ml).

Based on the results with the preferred model substrate *p*NP-β-d-glucopyranoside CIB12 exhibited high activities in the temperature range 30°C–60°C, which were all within 75% of its maximum activity at 50°C (Fig. 4C). The activity sharply decreased above 60°C, and no enzyme activity was observed at temperatures above 75°C. Compared to CIB12, CIB13 was active in a narrower temperature range (25°C–60°C) with the maximal activity observed at 50°C (Fig. 4D). Activity was not measurable at 70°C and above. Both enzymes showed a good thermostability at 40°C even after 5 h of incubation and retained 71.3% and 96.0% of activity for CIB12 and CIB13, respectively, after 1 h incubation at 50°C, but were not very stable at 50°C for longer >2 h incubations (Fig. 5A and B). Correspondingly, enzymes were denatured upon temperature increase as indicated by DSF, with melting points at 61°C and 64°C for CIB12 and CIB13, respectively (Fig. 5C and D), which is consistent with their optimal and maximal temperatures for activity. Obviously, GH1 enzymes from *Cuniculiplasma* did not exhibit optimal temperatures as high as their closest homologues from *T. acidophilum* (90°C) (Kim et al. 2009) and *Picrophilus oshimae* (70°C) (Murphy and Walsh 2019), but it should be noted that optimal temperatures of enzymes were well above the optimal temperatures for growth of *C. divulgatum* S5 (37°C–40°C) and much higher than average temperatures in the isolation site (Golyshina et al. 2016a). As reported earlier, the close relatedness of *Cuniculiplasma* spp. to thermophilic members of *Thermoplasmatales*, including *Thermoplasma* spp. and *Thermogymnomonas* spp. that are coclustering close to the Last *Thermoplasmatales* Common Ancestor root may point at a possible retention of some high-temperature-active enzymes inherited from their thermophilic ancestors (Bargiela et al. 2023), which could explain the discrepancies in temperature optima for enzyme activities and for growth.

### Salt and solvent tolerance

CIB12 and CIB13 activities were tested in the presence of NaCl concentrations varying from 0 to 4 M. The assays showed high NaCl tolerance of CIB12 activity with cellobiose: the activity remained high (80%–100%) at 0–1.5 M NaCl and gradually decreased to 50% at 4 M (Fig. 4E). CIB13 exhibited a steeper activity loss and showed ca 30% activity at 2.5 M and no activity at 4 M (Fig. 4F). Reports on salt tolerance are rare with some evidence where enzyme from GH1 shows salt tolerance such as enzyme from marine bacterium *Alteromonas* sp. L82 being active at 2 M NaCl (Sun et al. 2018) and enzyme from hyperthermophilic archaeon *Thermococcus* sp. retaining 100% activity across all NaCl concentrations (1–5 M) (Sinha and Datta 2016). Tween-20, a widely used nonionic detergent often employed for emulsion preparation, was tested at concentrations 0%–7%. For CIB12 (Fig. 4G), it was observed that enzyme activity was high between 0% and 1% Tween-20 after which it gradually decreased upon increase in Tween-20 concentration, and halved at 5% of Tween-20, and dropped to <2.5% at 6% of Tween. In contrast to that, CIB13 (Fig. 4H) enzyme activity was strongly promoted at 0.01%–1% of Tween-20 and then gradually decreased as the Tween-20 percentage in the reaction mixture increased. At maximal concentration tested, 7% of Tween-20, CIB13 retained >80% of its activity.

The kinetic parameters of purified CIB12 and CIB13 were investigated using the optimal reaction conditions and *p*NP-β-d-glucopyranoside, cellobiose, and lactose as variable substrates (Table 1). With *p*NP-β-d-glucopyranoside as substrate, CIB12 showed the *V_max_* at 0.61 U mg^−1^ with *K_M_* value of 0.5 mM, whereas CIB13 exhibited a slightly higher activity (0.93 U mg^−1^) but lower substrate affinity (*K_M_* 0.9 mM). Both enzymes also revealed similar catalytic efficiencies (*k*_cat_/*K*_M_) with *p*NP-ß-d-glucopyranoside, which were 1.1 mM^−1^ s^−1^ for CIB12 and 1.0 mM^−1^ s^−1^ for CIB13 (Table 1). With natural substrates (cellobiose and lactose), both enzymes revealed a higher affinity to cellobiose (*K*_M_ 26.2 mM and 13.6 mM) compared to lactose (*K*_M_ 50.7 mM and 100.5 mM) for CIB12 and CIB13, respectively. CIB12 demonstrated higher activity with natural substrates (*V_max_* 1.6–1.7 U mg^−1^) compared to CIB13 (*V_max_* 0.14–0.49 U mg^−1^), but the latter revealed a higher affinity to cellobiose (*K_M_* 13.6 mM) (Table 1). Most of the currently known archaeal GH1 β-glucosidases were characterized using chromogenic (*p*NP) substrates and usually preferred *p*NP-ß-d-glucopyranoside and *p*NP-β-d-galactopyranoside as substrates (Grogan 1991, Hei and Clark 1994, Ezaki et al. 1999, Srivastava et al. 2019), while Tkβgly from *T. kodakarensis* KOD1 had the highest catalytic efficiency (*k*_cat_/*K*_M_) with *p*NP-ß-d-fucopyranoside (Hwa et al. 2015). With natural substrates, the archaeal β-glucosidase Bgl1 from a thermophilic metagenome was found to be active with cellobiose and lactose (Schröder et al. 2014), PTO1453 from *P. torridus* hydrolysed lactose (Murphy and Walsh 2019), and cellobiose hydrolysis was demonstrated for GH1 β-glucosidases from *S. solfataricus* and *Pyrococcus kodakaraensis* (D’Auria et al. 1996, Ezaki et al. 1999).

**Table 1.** Kinetic parameters of purified CIB12 and CIB13 with various substrates at 30°C and optimal pH.

Protein	Substrates	*V_max_* (U mg^−1^)	*K_M_* (mM)	*k_cat_* (s^−1^)	k_cat_/K_M_ (mM^−1^ s^−1^)
CIB12	*p*NP-ß-d-glucopyranoside	0.61 ± 0.02	0.5 ± 0.1	0.57 ± 0.02	1.12 × 10^3^
	Cellobiose	1.60 ± 0.11	26.2 ± 4.1	1.50 ± 0.10	0.6 × 10^2^
	Lactose	1.70 ± 0.15	50.7 ± 9.4	1.60 ± 0.14	0.3 × 10^2^
CIB13	*p*NP-ß-d-glucopyranoside	0.93 ± 0.01	0.9 ± 0.1	0.87 ± 0.003	1.0 × 10^3^
	Cellobiose	0.49 ± 0.05	13.6 ± 4.5	0.46 ± 0.05	0.3 × 10^2^
	Lactose	0.14 ± 0.02	100.5 ± 26.6	0.13 ± 0.02	0.1 × 10

### Crystal structures of CIB12 and CIB13 reveal their active sites

**Figure 6.**
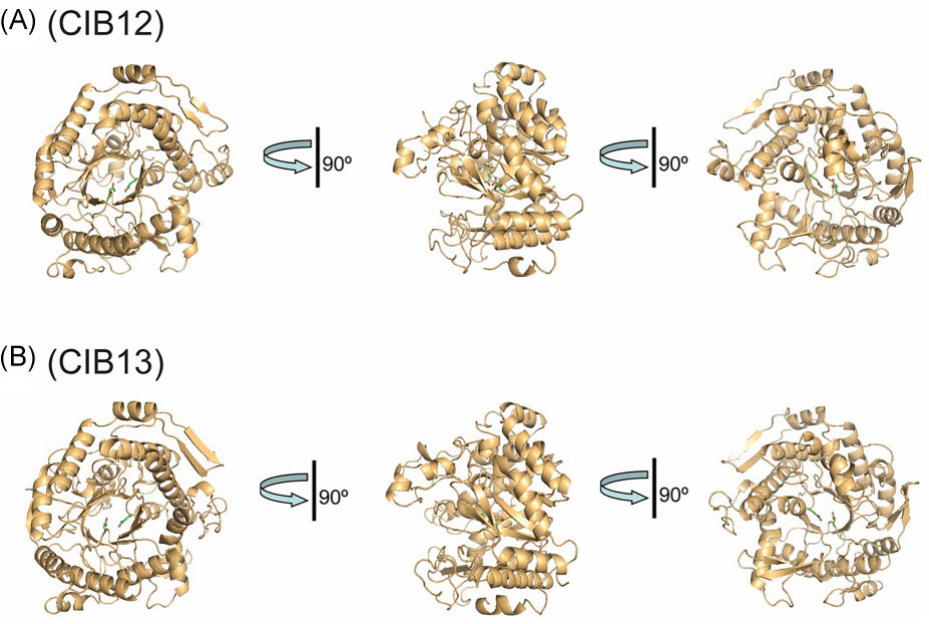
Crystal structures of CIB12 and CIB13: overall fold of protomers related by 90° rotations. The proteins are shown as ribbon diagrams with the active site glutamates shown as sticks.

Purified CIB12 and CIB13 were crystallized using the sitting-drop vapor diffusion method, and their crystal structures were determined to 2.55 Å and 2.22 Å resolution, respectively, by molecular replacement (Table S3, see the section “Materials and methods” for details). Both structures revealed a classical (β/α)_8_ TIM-barrel fold with a spherical shape, which is typical for GH1 enzymes and is one of the most common protein folds (Fig. 6) (Nagano et al. 2002). The inner wall of the TIM barrel includes the eight parallel β-strands, which are surrounded by eight α-helices (Fig. 6). In addition, the CIB12 structure contains two small antiparallel and one parallel β-sheets and six short α-helices, whereas CIB13 has four antiparallel β-sheets with two β-strands each and four α-helices outside the barrel structure (Fig. 6). Both CIB12 and CIB13 structures revealed the presence of two protomers per asymmetric unit forming a dimer with interacting residues located primarily on two C-terminal α-helices, one side β-strand, and connecting loops (Figs S4 and S5). Accordingly, the results of size-exclusion chromatography of purified CIB12 and CIB13 are suggesting that both proteins exist as a dimer in solution (75.9 kDa and 78.3 kDa with predicted monomeric masses 56.8 kDa and 56.1 kDa, respectively). The formation of protein dimers for CIB12 and CIB13 was also verified using the quaternary structure prediction server PDBePISA (buried areas 1117 Å^2^ and 1440 Å^2^, respectively). A Dali search for structurally homologous proteins in the PDB identified three GH1 structures with the β-glucosidase TsBGL from *Thermofilum* sp. ex4484_79 (PDB code 7F1N; Chen et al. 2021) as the best match for both CIB12 and CIB13 (Z-score 51.3 and 51.5, root mean square deviation 1.6 Å and 1.8 Å, sequence identity 40% and 41%, respectively). The other top hits include two GH1 β-glucosidases: the thermostable Bgl1317 from a soil metagenome (PDB code 6IER, 28% sequence identity to CIB12 and CIB13) and *aku*BGL from a sea slug (PDB code 8IN1, 24%–25% sequence identity) (Liu et al. 2019).

**Figure 7.**
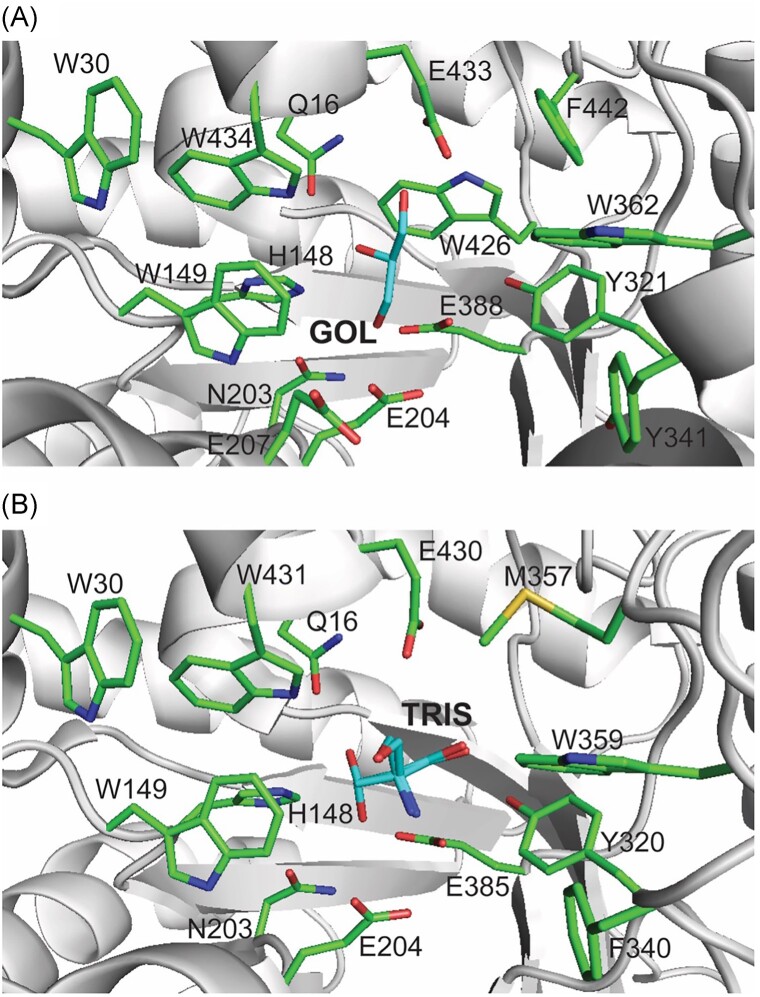
Crystal structures of CIB12 and CIB13: close-up view of the active sites. (A) CIB12 and (B) CIB13. The proteins are shown as ribbon diagrams with the side chains of active site residues shown as sticks including catalytic glutamates (E204 and E388 for CIB12 and E204 and E385 for CIB13). The bound ligand molecules are shown as sticks and labelled as GOL (glycerol) and TRIS (tris(hydroxymethyl)aminomethane).

The crystal structures of CIB12 and CIB13 also revealed their active sites with two catalytic glutamates located inside of the TIM barrel near its C-terminal side (Fig. 7). In the retaining catalytic mechanism of GH1 hydrolases, one of the two glutamates acts as an acid/base catalyst (Glu204 in CIB12 and CIB13) and the other acts as a nucleophile (Glu388 in CIB12 and Glu385 in CIB13) (Ketudat Cairns and Esen 2010). The cleavage of the glycosidic bond involves the formation of a covalent glucosyl-enzyme intermediate, which is then deglycosylated in a hydrolysis reaction via the attack of a water molecule producing the free enzyme and glucose. Both active sites show the presence of an additional area of electron density in each protomer located near the catalytic glutamates: Glu204 (3.9 Å) and Glu388 (2.8 Å) in CIB12 and Glu204 (3.5 Å) and Glu385 (2.8 Å) in CIB13 (Fig. 7, Fig. S5). In CIB12, this density was interpreted as glycerol (two molecules in the CIB12 dimer), whereas the CIB13 dimer contained one molecule of glycerol in one protomer and one molecule of tris(hydroxymethyl)aminomethane (Tris) in the second protomer (Fig. 7). The glycerol molecule in the CIB12 active site was also positioned close to the side chains of conserved His148 (3.1 Å), Trp149 (4.5 Å), Tyr321 (3.9 Å), Trp426 (4.1 Å), Glu433 (2.9 Å), and Trp434 (3.4 Å). Similarly, the Tris molecule in CIB13 was located near His148 (3.2 Å), Trp149 (3.7 Å), Tyr320 (2.6 Å), Trp423 (3.2 Å), Glu430 (3.4 Å), and Trp431 (3.2 Å). Additionally, both active sites contained several aromatic and charged residues, which might contribute to substrate binding including Gln16, Trp30, Asn203, Glu207, Tyr341, Trp362, and Phe442 in CIB12 and Gln16, Trp30, Asn203, Phe340, Met357, and Trp359 in CIB13. The crystal structure of CIB12 also revealed the presence of the disulfide bond Cys172–Cys212 located near the active site, which is not conserved in CIB13 and might contribute to a higher thermotolerance of CIB12 activity compared to CIB13 (Fig. 4). Surface charge analysis of both proteins revealed the presence of several positively or negatively charged patches with primarily negatively charged surfaces near the substrate-binding pocket (Fig. S6). Furthermore, analysis of surface hydrophobicity demonstrated the predominance of polar residues in both proteins with a few hydrophobic patches including the active site opening (e.g. Trp30/Trp30, Tyr321/Tyr320, Tyr341/Phe340, and Trp434/Trp431), which might be involved in cellulose binding (Fig. S6). Site-directed mutagenesis (alanine replacement) of CIB12 and CIB13 confirmed the important role of both catalytic glutamates (Glu204/Glu388 and Glu204/Glu385, respectively) and several other active site residues (His148 and Trp426 in CIB12, Tyr320 and Trp431 in CIB13) for enzymatic activity of both proteins, whereas the Asn203Ala and Phe340Ala mutant proteins retained detectable activity (Fig. S7).

### Physiological and ecological roles of β-glucosidases: growth of *C. divulgatum* S5 is enhanced by cellobiose

**Figure 8.**
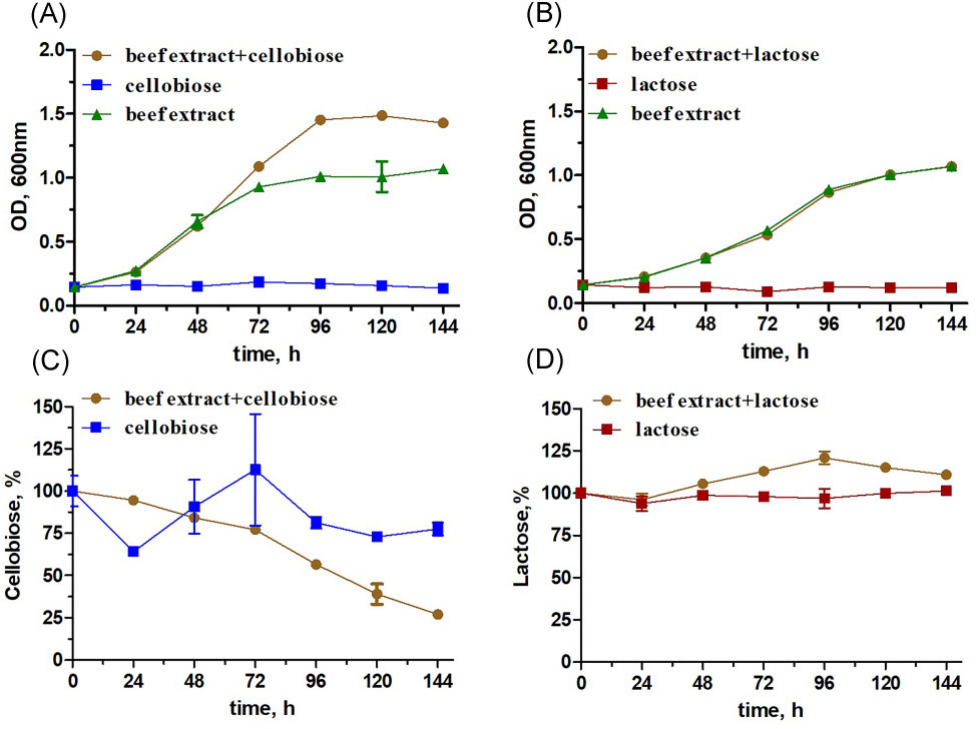
Growth of *C. divulgatum* cultures with beef extract and consumption of added disaccharides. (A and B), Growth of *C. divulgatum* cultures (OD, 600 nm) in DSMZ medium 88 (see the section “Materials and methods”) with/without addition of beef extract (0.3% w/v), cellobiose (0.1% (w/v), and/or lactose (0.1%). (C and D), Residual concentrations of cellobiose and lactose during growth of *C. divulgatum* cultures, as revealed by HPLC analysis of culture supernatants.

Our work on characterization of GH1 enzymes, and their ability to hydrolyse cellobiose and lactose pointed at their potential function *in vivo*, namely utilization of these compounds for growth. Cultivation of *C. divulgatum* S5 with 3 mM (or 0.1%) cellobiose and lactose in the presence of beef extract (0.3%) revealed significant, up to 50%, increase in biomass yields with cellobiose (Fig. 8), but not lactose. Cellobiose was indeed consumed during the growth, with its residual concentration in the medium being ~25% at the end of the experiment. No growth of the strain was observed with either disaccharide alone, i.e. without beef extract, while concentrations of these disaccharides remained at the initial levels. Simultaneously, no measurable levels of glucose were detected in HPLC chromatograms of supernatants of cellobiose-spiked cultures, suggesting its hydrolysis appears inside the cells, consistently with the predicted intracellular localization of tested glycosidases and with our previous study that showed no growth stimulation by glucose (Golyshina et al. 2016b). In the *C. divulgatum* S5 genome, both CIB12 and CIB13 do not appear to be associated with any operon and are encoded by single genes without signal peptides suggesting that they are intracellular proteins (Golyshina et al. 2016b). From the proteomic data obtained earlier (Bargiela et al. 2020), both proteins were detectable, albeit at low basal levels, correspondingly, 0.017% and 0.014% of the total proteome under standard cultivation conditions, i.e. without addition of glycosidic substrates. In a broader context, all *Thermoplasmatales* cultured so far, do rely on peptide-, or oligopeptide substrates-based diets. Yeast extract, tryptone, and/or beef extract are hence essential ingredients in their media, however some thermophilic members of this order produce higher biomass yields in presence of mono-, or disaccharides (Huber and Stetter 2006). The apparent ability to utilize intermediates of the breakdown of cellulosic compounds, previously not reported in *Cuniculiplasma* spp., points at their extended potential to colonize niches where they are neighbouring the primary (polysaccharide) producing organisms, e.g. *Dunaliella* sp., *C. acidophila, E. mutabilis*, and *Bryopsida* sp. (Distaso et al. 2022). In photoheterotrophs, the ability to degrade cellulose by secreted endo-β-1,4-glucanases to cellobiose, with its consequent transport and assimilation was earlier demonstrated for *Chlamydomonas* spp. (Blifernez-Klassen et al. 2012). The ability to hydrolyse cellulose is also known for heterotrophic acidophilic/acidotolerant bacteria, for example, in *Acidisoma* spp. from *Rhodospirillales* (Mieszkin et al. 2021), *Alicyclobacillus* spp. (Kusube et al. 2014), *Acidothermus* spp. (Mohagheghi et al. 1986), and acidobacteria (Kielak et al. 2016, Gonzàlez et al. 2020), to name just a few examples. This points at the intrinsic microbial enzymatic potential to degrade cellulosic polymers in acidic systems to provide oligosaccharides, including cellobiose, to noncellulolytic organisms. Thus, *Cuniculiplasma* spp., and likely, other *Thermoplasmatales* as well, may become a part of a wider cellulose-degrading community and significantly contribute to the carbon cycling thanks to their intracellular cellobiohydrolases.

## Conclusion

In this work, two representatives of glycosidases of GH1 family from *C. divulgatum* were characterized. Both CIB12 and CIB13 were active against cellobiose and lactose but showed substrate preference to cellobiose, and therefore were identified as cellobiohydrolases, EC 3.2.1.21. Both enzymes exhibited activities at broad pH ranges with slightly acidic optima and at temperatures as high as 60°C, with the optimum at 50°C. We suggest the robust glycosyl hydrolase activities of these enzymes at elevated temperatures may have been inherited from their thermophilic ancestors, and have been retained even after the colonization of colder environments. Furthermore, their ability to hydrolyse the cellobiose, the intermediate product of degradation of cellulosic materials is a novel trait identified in *C. divulgatum* and in acidophilic archaea inhabiting acid mine drainage sites. This trait expands the range of substrates used by *C. divulgatum*, which was assumed to be limited to oligo- and polypeptides. This also implies an increased scale of their involvement into the carbon cycling, suggesting greater ecological significance of these archaea in acidic environments.

## Supplementary Material

fiae114_Supplemental_File
