# Supplementary material for: Moderately thermostable GH1 β-glucosidases from hyperacidophilic archaeon Cuniculiplasma divulgatum S5
Source: FEMS Microbiol Ecol. 2024 Aug 10;100(9):fiae114. doi: 10.1093/femsec/fiae114 (PMC11376072; doi:10.1093/femsec/fiae114)
Supplement: fiae114_Supplemental_File [file fiae114_supplemental_file.docx]

Supplementary Figures and Tables

Khusnutdinova A.N. et al. **Moderately thermostable GH1 β-glucosidases from hyperacidophilic archaeon *Cuniculiplasma divulgatum* S5**

**A**


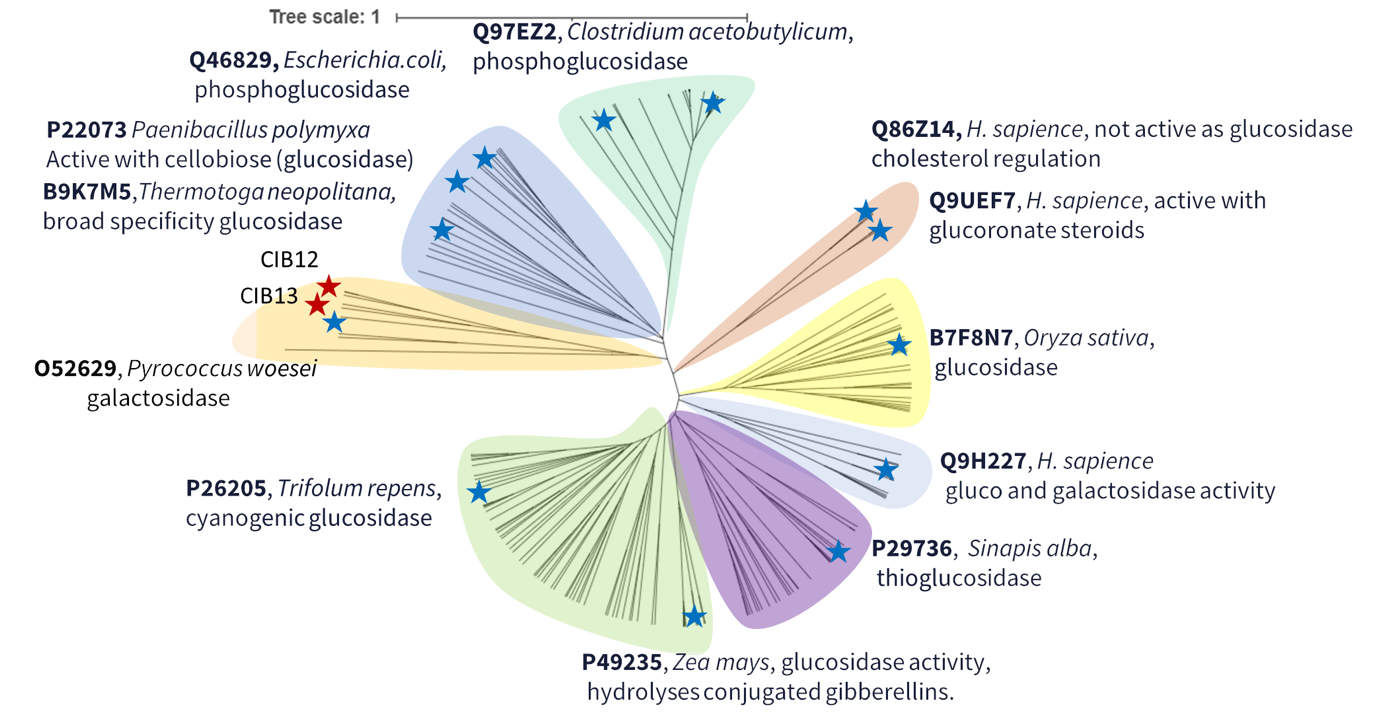


**Q86Z14**, *H. sapiens*, not active as glucosidase cholesterol regulation

**Q9UEF7**, *H. sapiens*, active with glucoronate steroids

**Q9H227**, *H*. *sapiens* glucosidase and galactosidase activity

**B**


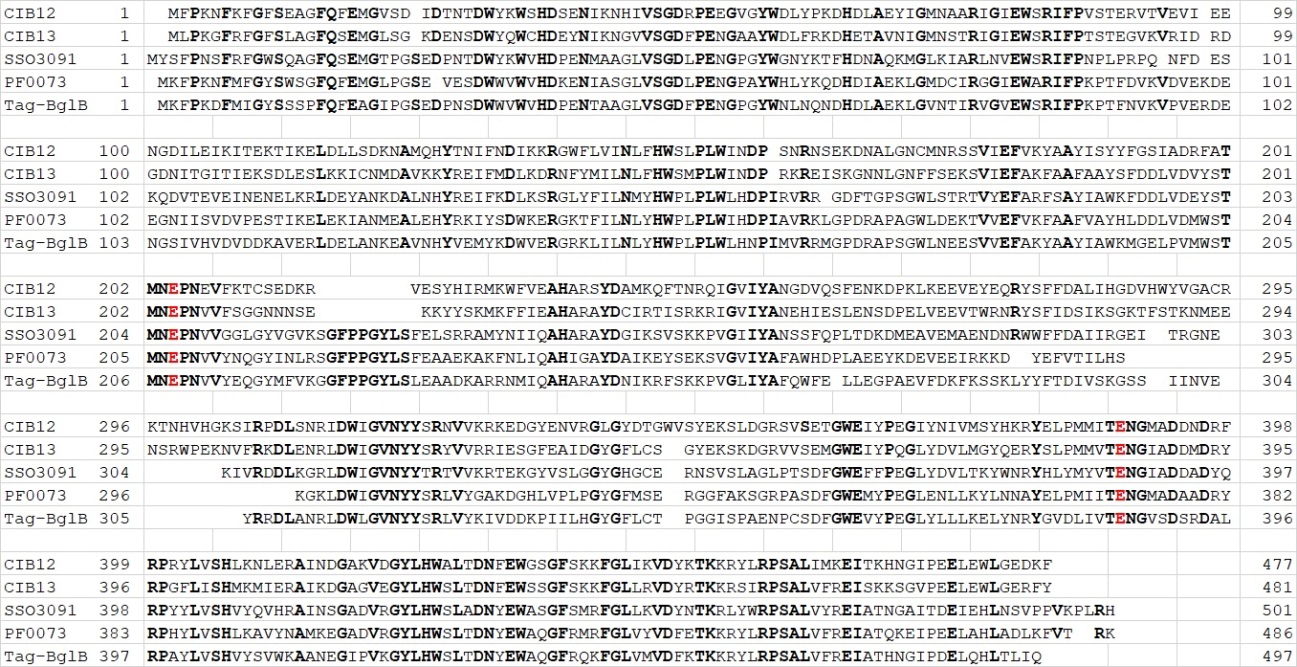


**Figure S1.** (**A)** Phylogenetic analysis of the GH1 family using the UniProt IPR001360 sequences. Blue stars indicate characterised enzymes from different organisms, red stars denote CIB12 and CIB13. **(B)** Amino acid sequence alignment of CIB12 and CIB13 with the biochemically characterized GH1 glycoside hydrolases SSO3091 from *Saccharolobus solfataricus* (Uniprot ID P22498), PF0073 from *Pyrococcus furiosus* (E7FHY4), and Tag-BglB from *Thermosphaera aggregans* (Q9YGA8). Conserved residues are shown in bold font, whereas the catalytic glutamates are coloured red.


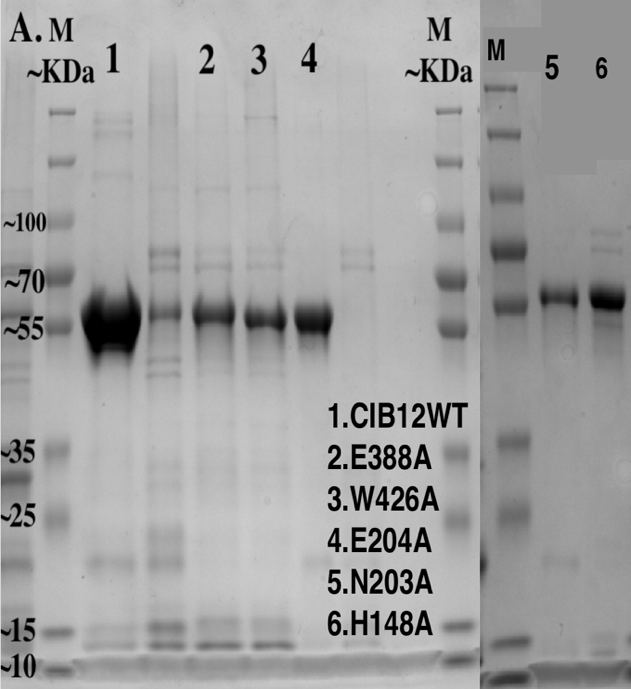


**
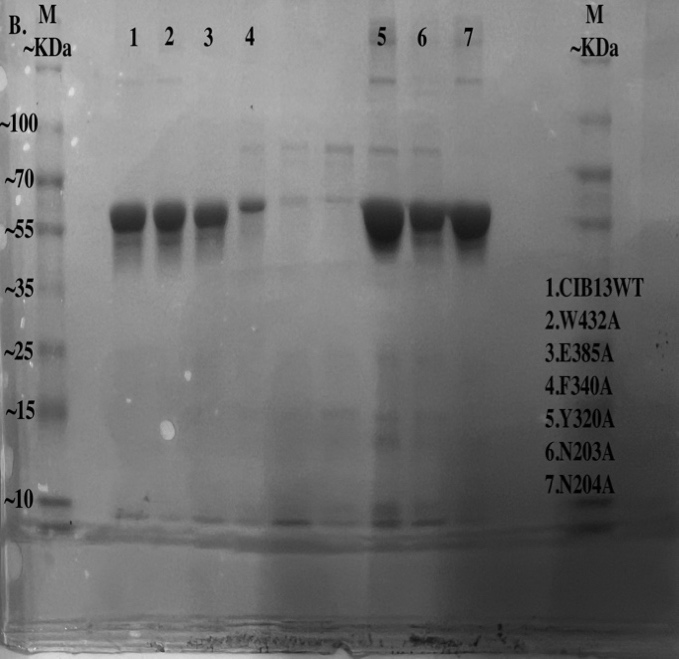
**

**Figure S2.** SDS-PAGE analysis of purified wild type and mutant CIB12 and CIB13 proteins. (A), CIB12 (H148A, E388A, W426A, E204A, N203A, Y321A, Y341A, W362A); (B), CIB13 (W432A, E385A, F340A, Y320A, N204A, N203A, W426A). Lane M, protein Mw markers, 10-250 kDa; protein load: 2 μg/well.


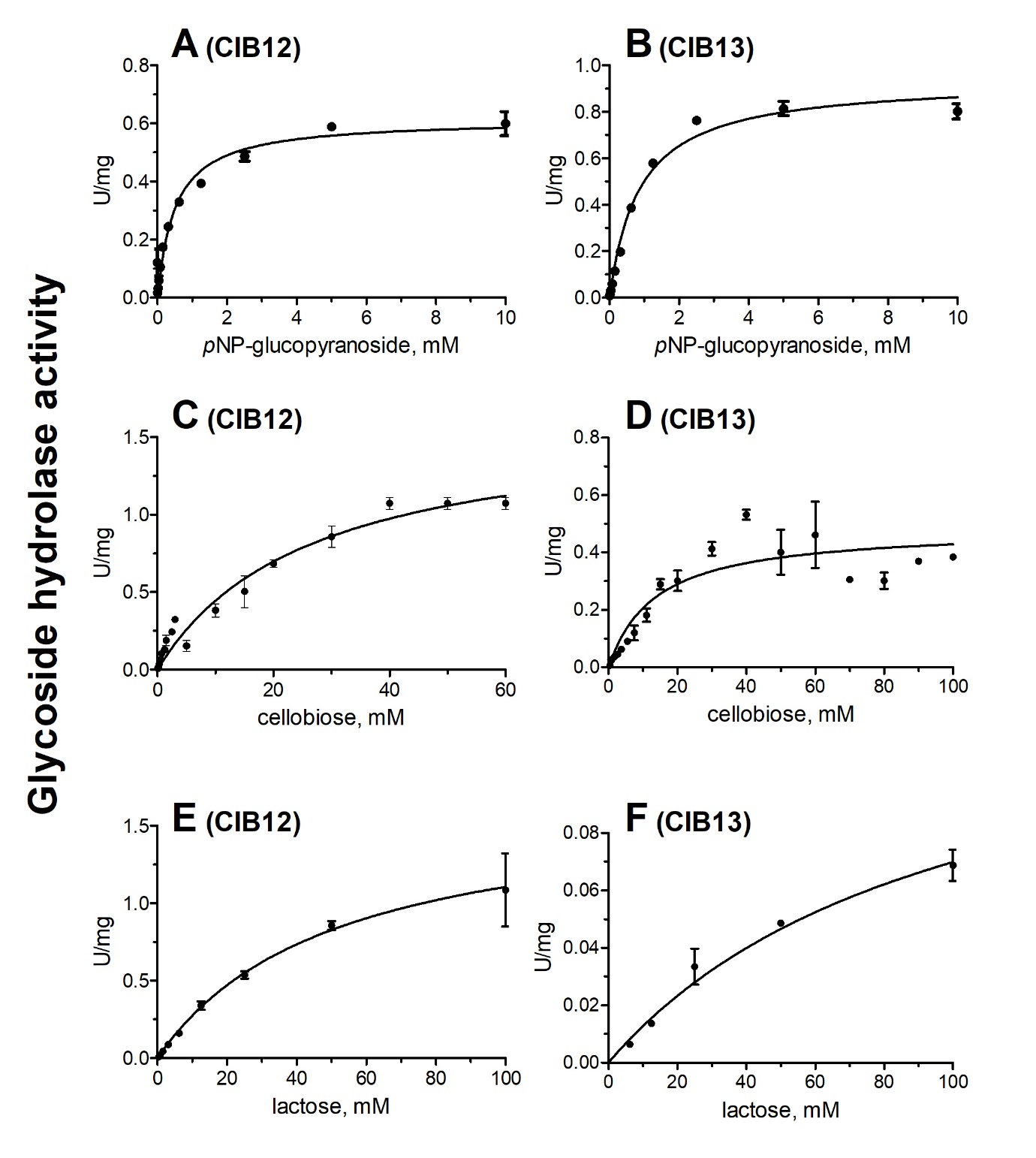


**Figure S3.** Glycoside hydrolase activity of purified CIB12 and CIB13 as a

function of substrate concentration. (A, B), *p*NP-β-D-glucopyranoside as substrate; (C, D), cellobiose as substrate; (E, F), lactose as substrate. Reaction mixtures contained 50 mM MES buffer (pH 5.0), indicated substrates, and 5 μg of enzyme (incubation at 30 °C, 2 h for A and B, 6 h for C, D, E, and F).


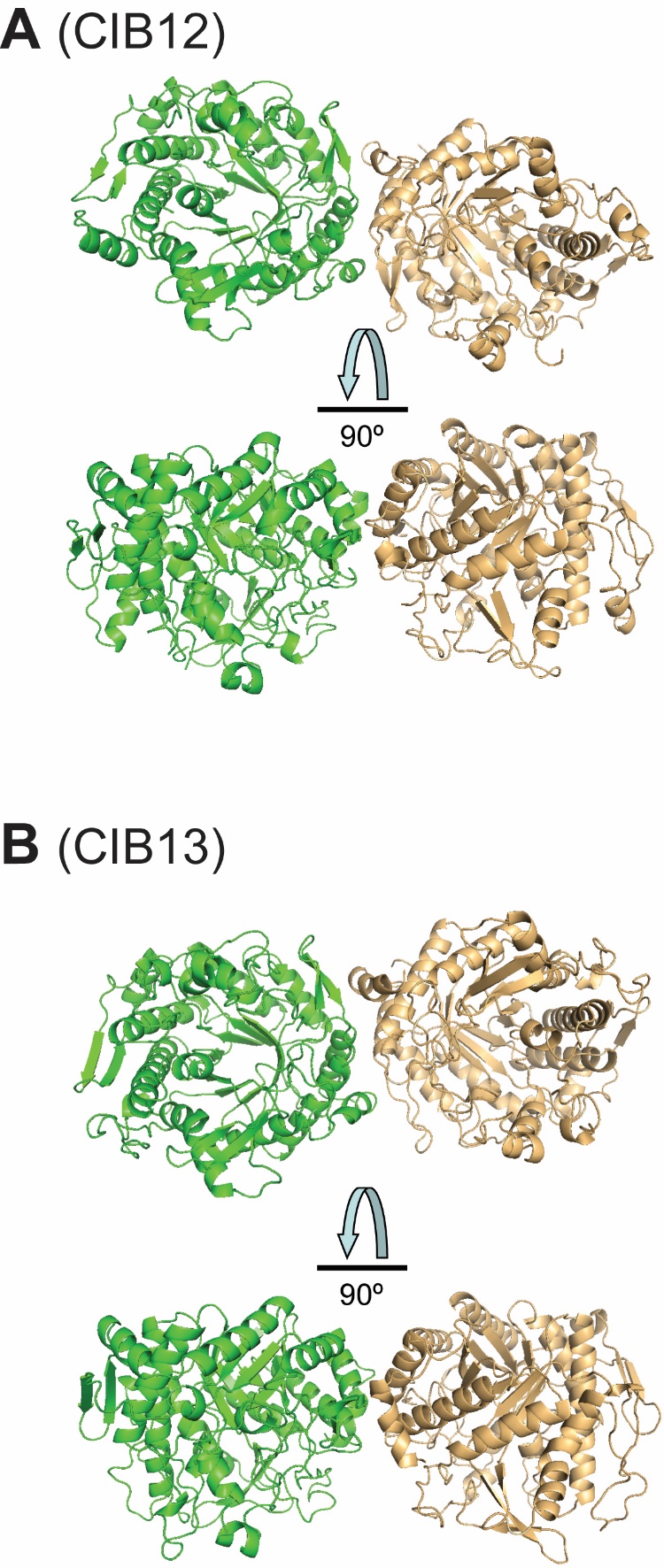


**Figure S4.** Crystal structures of CIB12 and CIB13: overall view of dimers related by 90° rotations. The protein subunits are shown as ribbon diagrams with different colours (green and orange).


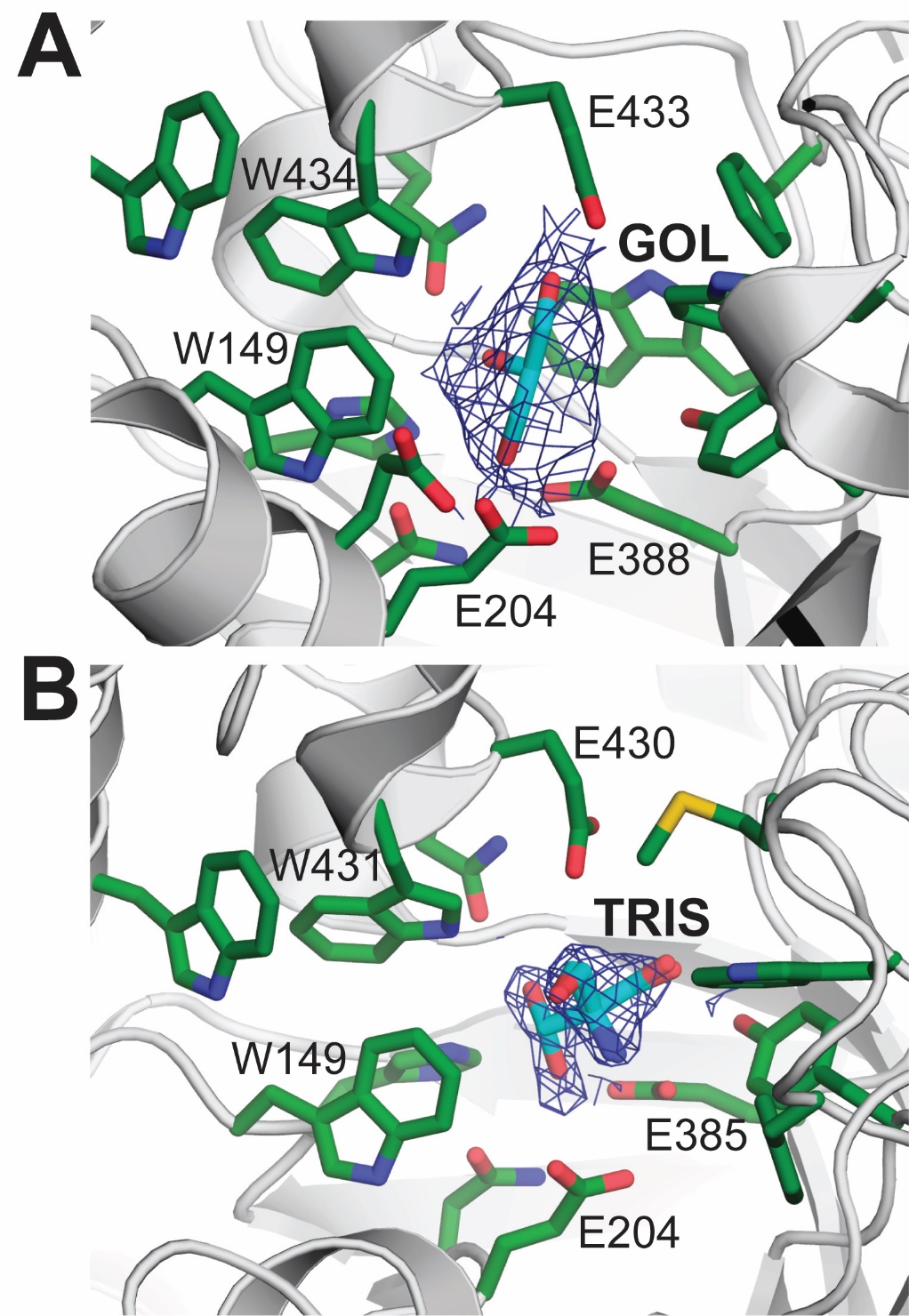


**Figure S5**. Active sites of CIB12 and CIB13 showing the bound ligand molecules: (A), CIB12 with bound glycerol (GOL); CIB13 with bound (tris(hydroxymethyl)aminomethane) (TRIS). The proteins are presented as grey ribbon diagrams with amino acid side chains shown as sticks with green carbons and bound ligand molecules as sticks with cyan carbons. Density features shown are 2F_o_-F_c_ maps contoured at 1.0 σ.


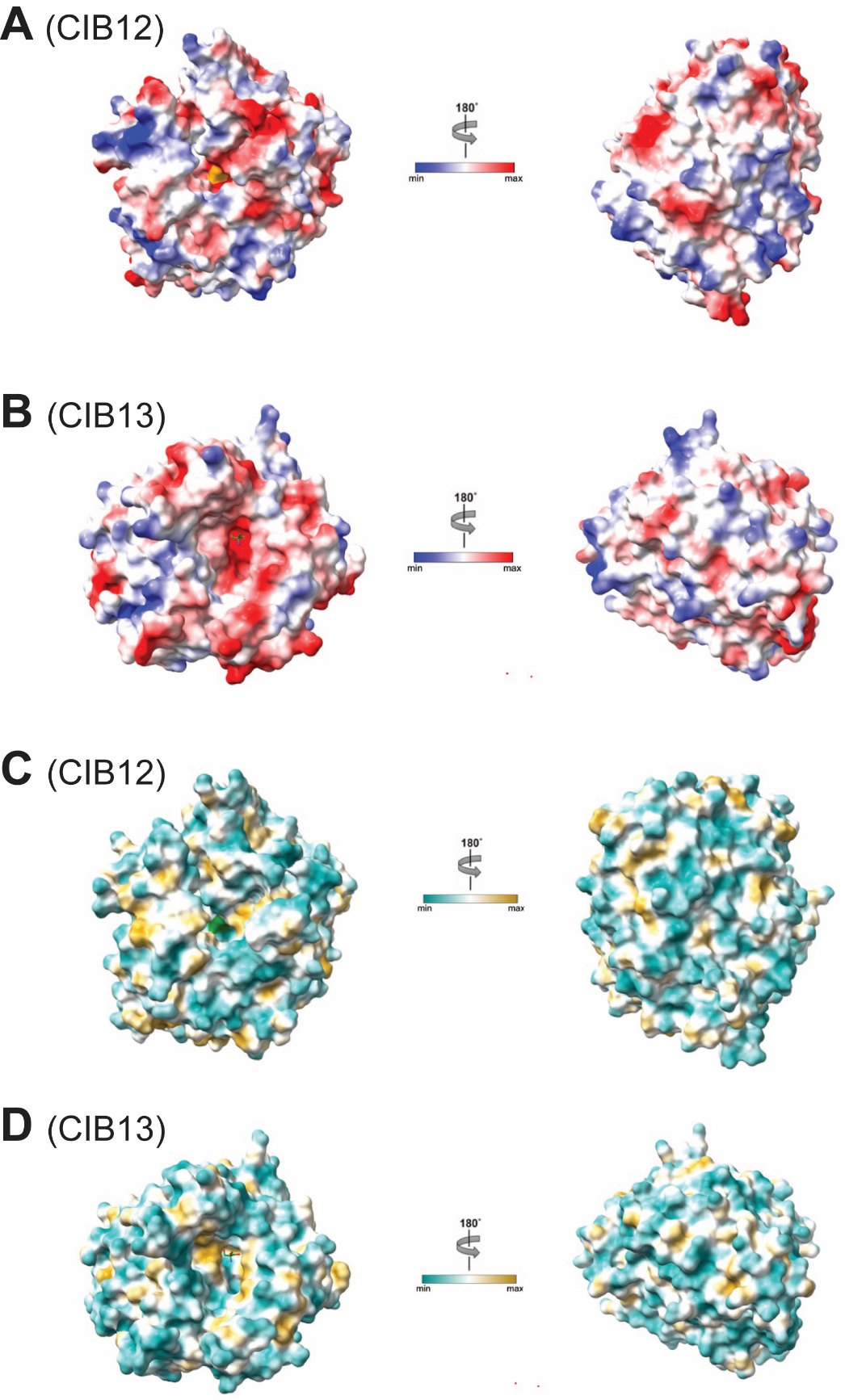


**Figure S6.** Structural analysis of CIB12 and CIB13: surface charge and hydrophobicity. Surface presentation of CIB12 (A, C) and CIB13 (B, D) protomers showing the distribution of surface charge (A, B) and hydrophobicity (C, D) of solvent accessible protein residues. Distribution of surface potential (Coulombic charge) has been shown as a red (negatively charged) to white (nearly neutral) to blue (positively charged) gradient, whereas the distribution of surface hydrophobicity is shown as a cyan (polar) to yellow (hydrophobic) gradient. The protomers are presented in two views related by a 180° rotation with the scale bars showing coloring schemes of surface exposed residues.


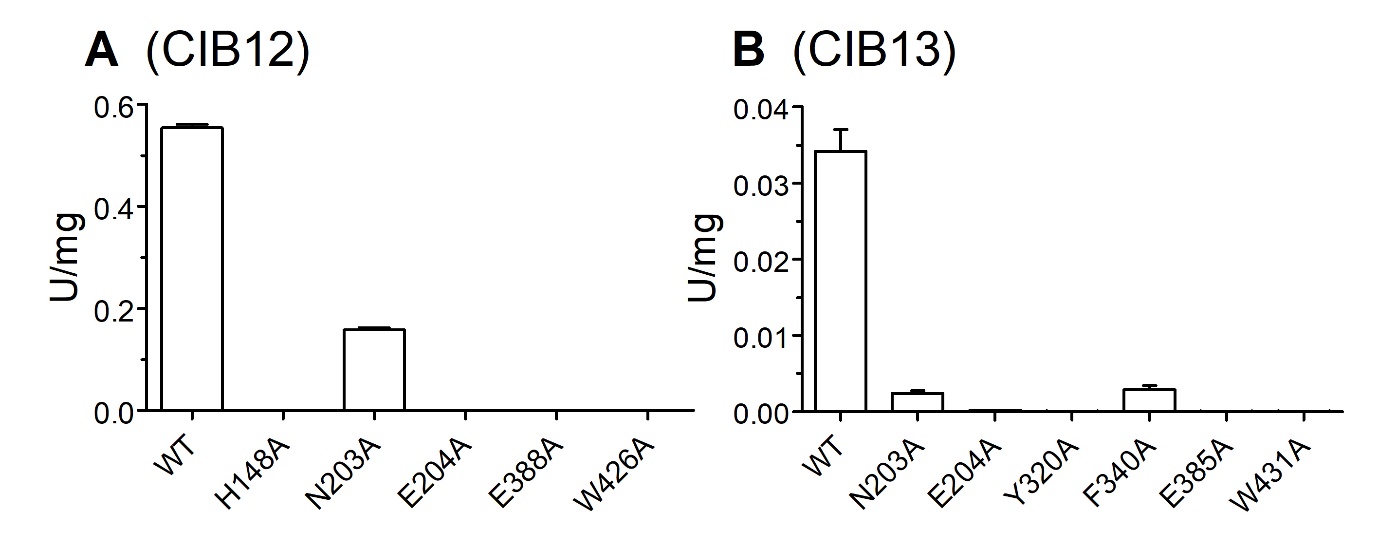


**Figure S7.** Site-directed mutagenesis of CIB12 and CIB13: glycoside hydrolase activity of purified wild type and mutant proteins with 2 mM *p*NP-β-D-glucopyranoside.

**Table S1.** The list of chromogenic (*p*NP-) substrates used in this study.

| 1. *p*NP-α-D-arabinopyranoside | 12. *p*NP-α-D-maltohexaoside |
| --- | --- |
| 2. *p*NP-β-D-arabinofuranoside | 13. *p*NP-α-D-maltopyranoside |
| 3. *p*NP-β-D-cellobiopyranoside | 14. *p*NP-β-D-maltopyranoside |
| 4. *p*NP-α-L-fucopyranoside | 15. *p*NP-α-D-mannopyranoside |
| 5. *p*NP-α-D-galactopyranoside | 16. *p*NP-β-D-mannopyranoside |
| 6. *p*NP-β-D-galactopyranoside | 17. *p*NP-β-D-ribofuranoside |
| 7. *p*NP-β-D-galactopyranoside tetra-acetate | 18. *p*NP-α-L-rhamnopyranoside |
| 8. *p*NP-α-D-glucopyranoside | 19. *p*NP-α-D-xylopyranoside |
| 9. *p*NP-β-D-glucopyranoside | 20. *p*NP-β-D-xylopyranoside |
| 10. *p*NP-α-D-glucuronide | 21. *p*NP-N-acetyl-β-D-glucosaminide |
| 11. *p*NP-β-D-lactopyranoside |  |

**Table S2.** The list of natural glycoside hydrolase substrates used in this study.

| 1. acarbose | 11. γ-cyclodextrin |
| --- | --- |
| 2. amylose | 12. D-xylan |
| 3. amylopectin (maize) | 13. gum arabic |
| 4. agarose | 14. lactose |
| 5. cellobiose | 15. maltotriose |
| 6. chitin | 16. pectin |
| 7. dextrin (potato) | 17. pullulan |
| 8. carboxymethylcellulose (CMC) | 18. starch (potato) |
| 9. α-cyclodextrin | 19. trehalose |
| 10. ß-cyclodextrin |  |

**Table S3.** X-ray crystallographic statistics for the structures of CIB12 and CIB13.

| Structure | CIB12 | CIB13 |
| --- | --- | --- |
| PDB code | 8U7F | 8U7G |
| Data collection |  |  |
| Space group | C2 | P2_1_ |
| Cell dimensions  *a*, *b, c* (Å)  α, β, γ, (°) | 193.21, 51.81, 128.79  90, 108.3, 90 | 52.80, 102.19, 97.54  90, 104.7, 90 |
| Resolution, Å | 25.00 – 2.55 | 30.00 – 2.22 |
| R*_merge_^a^*  R*_pim_* | 0.093 (0.581)^b^  0.047 (0.289) | 0.061 (0.310)  0.036 (0.215) |
| *I* / σ(*I)* | 18.33 (1.13) | 16.88 (1.93) |
| Completeness, % | 95.2 (81.5) | 98.0 (81.8) |
| Redundancy | 4.9 (4.7) | 3.6 (2.7) |
| Refinement |  |  |
| Resolution, Å | 24.93 – 2.55 | 29.63 – 2.21 |
| No. of unique reflections:  working, test | 38069, 1902 | 49054, 2007 |
| *R*-factor/free *R­*-factor^c^ | 22.0/26.0 (32.8/35.7) | 17.9/21.9 (26.1/34.2) |
| No. of refined atoms, molecules  Protein  Solvent  Water | 7924, 2  12  107 | 7936, 2  22  670 |
| *B*-factors  Protein  Solvent  Water | 64.4  55.1  52.7 | 41.5  41.6  43.7 |
| r.m.s.d.  Bond lengths, Å  Bond angles, ° | 0.003  0.553 | 0.002  0.498 |
|  |  |  |

^a^*R*_merge_ = Σ_h_Σ_i_|*I*_i_(*h*) - 〈*I*(h)〉/Σ_h_Σ_i_I_i_(*h*), where *I*_i_(*h*) and 〈*I*(*h*)〉 are the *i*th and mean measurement of the intensity of reflection *h*.

^b^Figures in parentheses indicate the values for the outer shells of the data.

^c^*R* = Σ|F_p_^obs^ – F_p_^calc^|/ΣF_p_^obs^, where F_p_^obs^ and F_p_^calc^ are the observed and calculated structure factor amplitudes, respectively.
